# Constitutional de novo and postzygotic mutations in isolated cases of cerebral cavernous malformations

**DOI:** 10.1002/mgg3.256

**Published:** 2016-12-20

**Authors:** Matthias Rath, Stefanie Spiegler, Neetika Nath, Konrad Schwefel, Nataliya Di Donato, Johannes Gerber, G. Christoph Korenke, Yorck Hellenbroich, Ute Hehr, Stephanie Gross, Ulrich Sure, Barbara Zoll, Eberhard Gilberg, Lars Kaderali, Ute Felbor

**Affiliations:** ^1^ Department of Human GeneticsUniversity Medicine Greifswald and Interfaculty Institute of Genetics and Functional GenomicsUniversity of GreifswaldGreifswaldGermany; ^2^ Institute for BioinformaticsUniversity Medicine GreifswaldGreifswaldGermany; ^3^ Institute for Clinical GeneticsFaculty of Medicine Carl Gustav CarusTU DresdenDresdenGermany; ^4^ Department of NeuroradiologyUniversity Hospital Carl Gustav CarusDresdenGermany; ^5^ Department of NeuropaediatricsChildren's Hospital OldenburgOldenburgGermany; ^6^ Institute of Human GeneticsUniversity of LübeckLübeckGermany; ^7^ Center for and Institute of Human GeneticsUniversity of RegensburgRegensburgGermany; ^8^ Department of NeuropediatricsJustus‐Liebig‐UniversityGießenGermany; ^9^ Department of NeurosurgeryUniversity Hospital EssenUniversity of Duisburg‐EssenEssenGermany; ^10^ Institute of Human GeneticsGeorg August UniversityGöttingenGermany

**Keywords:** *CCM1*, *CCM2*, *CCM3*, cerebral cavernous malformation, de novo mutation, deep sequencing, postzygotic mutation

## Abstract

**Background:**

Cerebral cavernous malformations (CCM) are vascular lesions of the central nervous system that can be found in sporadic or autosomal dominantly inherited forms and manifest with headaches, seizures, and hemorrhagic stroke. The precise proportion of de novo mutations in the *CCM1*,*CCM2*, and *CCM3* genes remains unknown.

**Methods:**

We here present a series of six trios with de novo mutations that have been analyzed by amplicon deep sequencing to differentiate between constitutional and postzygotic mutations.

**Results:**

In one case, allelic ratios clearly indicated mosaicism for a *CCM3* splice site mutation found in blood and buccal mucosa of a 2‐year‐old boy with multiple CCMs. The remaining five de novo mutations proved to be constitutional. In addition to three *CCM3*, two *CCM1*, and one *CCM2* de novo point mutations, a deletion of the entire *CCM3* gene was identified in an index case that most likely originated from an early postzygotic event. These are the first high‐level mosaic mutations reported in blood samples of isolated CCM cases.

**Conclusion:**

Our data demonstrate that de novo mutations in *CCM1‐3* might be more frequent than previously thought. Furthermore, amplicon deep sequencing is useful to discriminate between patients with constitutional and postzygotic mutations, and thereby improves genetic counseling.

## Introduction

Cerebral cavernous malformations (CCM) are low‐flow vascular lesions that are located in the brain or the spinal cord and occur with a prevalence of about 1:650 in the general population (Morris et al. 2009). The familial form (OMIM 116860, 603284, 603285) accounts for up to 20% of all cases and is inherited in an autosomal dominant manner. Heterozygous loss‐of‐function mutations in *CCM1* (*KRIT1;* OMIM *604214), *CCM2* (Malcavernin/OSM; *607929), and *CCM3* (PDCD10; *609118) have been associated with CCM.

Due to an impaired function of tight and adherens junctions, CCMs are prone to leakiness and rupture. Clinical symptoms mostly arise from recurrent bleedings and may include headaches, seizures, and hemorrhagic stroke. Nevertheless, CCMs are characterized by an incomplete disease penetrance with up to 30–45% of all patients remaining asymptomatic (Denier et al. 2006). For index cases with multiple CCMs or a positive family history, genetic testing is recommended. With the stepwise use of conventional sequencing and multiplex ligation‐dependent probe amplification (MLPA), high mutation detection rates have been achieved. Following the established criteria, pathogenic variants can be found in up to 87% of familial and 57% of isolated cases (Spiegler et al. 2014). De novo mutations have occasionally been reported previously, but their precise prevalence is still unknown (Bergametti et al. 2005; Liquori et al. 2006; Stahl et al. 2008; Riant et al. 2013; Cigoli et al. 2014).

In the study presented here, next‐generation deep sequencing was used to answer the question whether de novo point mutations identified in six out of 60 isolated CCM cases have arisen as postzygotic mutations or may be present as constitutional variants. To the best of our knowledge, we herein describe the first high‐level mosaicism reported in a CCM patient.

## Materials and Methods

### Probands and DNA samples

Isolated cases of our previously published (Stahl et al. 2008; Spiegler et al. 2014) and consecutively extended cohort were included in this study (see Table 1). Study design was approved by the ethics committee of the University Medicine Greifswald (registration number: BB 047/14). In addition, all probands participated with written informed consent according to the German Gene Diagnostics Act. Genomic DNA was isolated from blood lymphocytes using NucleoSpin^®^ Blood Kit (Macherey‐Nagel, Düren, Germany) or buccal mucosa using Gentra Puregene Buccal Cell Kit (Qiagen, Hilden, Germany). Paternity was confirmed for all de novo cases using PowerPlex 16 System (Promega, Mannheim, Germany).

**Table 1:** De novo mutations found in isolated cases harboring multiple CCMs

Proband					Mutation					
No.	Sex	Age at onset	Clinical symptoms and MRI findings	Reference of previously described cases	Gene	Nucleotide change^a^	Alternate allele read frequency in ADS^b^	*Z*‐score	*P*‐value after BHC	First description of the mutation
P1	M	2y	Acute left‐sided hemiparesis due to hemorrhage of a brainstem CCM, multiple supra‐ and infratentorial CCMs on MRI scans	Previously unpublished	*CCM3*	c.474+5G>A	35% (1865×)	−2.86	0.026	Liquori et al. (2006)
P2	F	1y	Recurrent bleedings of a brainstem CCM with deficits of multiple cranial nerves, two additional supratentorial CCMs	Previously unpublished	*CCM2*	c.563_564dupGG	41% (1700×)	−1.52	0.260	Novel
P3	M	2y	Multiple CCMs identified as incidental finding on MRI scans of a boy with a mental retardation	Previously unpublished	*CCM3*	c.395+1G>A	46% (287×)	−0.40	0.829	D'Angelo et al. (2011)
P4	M	14y	Multiple CCMs with epileptic seizures	Spiegler et al. (2014)	*CCM1*	c.1660_1678del	44% (1040×)	−0.84	0.597	Spiegler et al. (2014)
P5	F	15y	Proximal paresis of the left arm and right‐sided dysesthesia, epileptic seizures, multiple CCMs on MRI scans	Stahl et al. (2008)	*CCM1*	c.2143‐2A>G	46% (418×)	−0.40	0.691	Stahl et al. (2008)
P6^c^	F	19y	Epileptic seizures	Spiegler et al. (2014)	*CCM3*	c.391delA	55% (93×)	1.61	0.320	Spiegler et al. (2014)
P7	M	32y	Obstructive hydrocephalus with headaches and an acute brainstem herniation due to a large CCM of the left cerebellar hemisphere	Previously unpublished	*CCM3*	Deletion of the entire gene	n.d.	n.d.	n.d.	Bergametti et al. (2005)

a *CCM1* (LRG_650t1; ENST00000394507.5), *CCM2* (LRG_664t2; ENST00000258781.10), *CCM3* (NM_007217.3; ENST00000392750.6).

b Calculated as the percentage of variant reads from the total number of reads (total coverage in brackets); y, year; n.d., not done; BHC, Benjamini–Hochberg correction.

c The daughter of P6 also carries the familial *CCM3* mutation and presented with multiple, symptomatic CCMs (epileptic seizures and acute CCM hemorrhage) at the age of 12 months.

### Mutation analyses

Coding exons and adjacent splice sites of *CCM1* (LRG_650t1; ENST00000394507.5), *CCM2* (LRG_664t2; ENST00000258781.10), and *CCM3* (NM_007217.3; ENST00000392750.6) were sequenced as described elsewhere (Spiegler et al. 2014). DNA mutation numbering is based on cDNA sequence with +1 corresponding to the A of the ATG translation initiation codon (http://varnomen.hgvs.org/). SALSA MLPA Kits P130 & P131 were used for the detection of copy number variations (MRC Holland, Amsterdam, Netherlands).

### Digital PCR

Absolute quantification of genomic DNA was assessed using the QuantStudio^®^ 3D Digital PCR System (Thermo Fisher Scientific, Waltham, MA, USA) with an Universal Probe Library assay for *CCM3* (Roche, UPL#30, Primer left: 5′‐AGCAGAAGAGGTCTAGGGTCAC‐3′, Primer right: 5′‐CTCCTCTTCCGCCCGTAG‐3′) and the *TBP* gene as reference (Roche, UPL#3, Primer left: 5′‐CAGCCGTTCAGCAGTCAAC‐3′, Primer right: 5′‐TGTGAGTGGAAGAGCTGTGG‐3′).

### Deep sequencing and data analysis

Genomic regions covering de novo mutations were PCR‐amplified with the high‐fidelity PrimeSTAR GXL (Takara Clontech, Saint‐Germain‐en‐Laye, France) or KAPA HiFi polymerase (Kapa Biosystems, London, UK). Primer sequences are available upon request. Following purification of the amplicons with Agencourt^®^ AMPure^®^ XP beads (Beckman Coulter, Pasadena, CA, USA), Nextera XT Kit was used for library preparation (Illumina^®^, San Diego, CA, USA).

Target enrichment of all exons of *CCM1‐3* using a custom‐made HaloPlex panel (Agilent, Santa Clara, CA, USA) was performed as previously described (Kohda et al. 2016). All libraries were sequenced on a MiSeq sequencer with 2 × 250 cycles using Reagent Kit v3 (Illumina^®^). The SeqNext software (JSI Medical Systems, Ettenheim, Germany) was used for read mapping to the reference genome hg19 and variant calling. *Z*‐scores for allelic ratios of variants were calculated as previously described (Acuna‐Hidalgo et al. 2015). Statistical significance was assumed with a critical value of *P* < 0.05 after Benjamini–Hochberg correction.

## Results

### Characteristics of isolated CCM cases

Since 2005, a total of 60 isolated cases with multiple CCMs were analyzed (Stahl et al. 2008; Spiegler et al. 2014). Conventional Sanger sequencing and MLPA analysis identified pathogenic variants in 34 individuals (56%). Despite our best efforts, DNA samples of both parents were available for only eight of all mutation‐positive probands as many of the asymptomatic parents were unavailable or had decided against predictive testing. De novo mutations could be verified in six cases based on conventional sequencing of parental lymphocyte DNA (P1–P6, Table 1). Three *CCM3*, two *CCM1*, and one *CCM2* de novo point mutations were identified. Nonpaternity was excluded for these cases. Either one parent of further two probands proved to be an asymptomatic carrier of a familial mutation (data not shown).

The clinical symptoms of de novo CCM cases varied from multiple asymptomatic CCMs identified as incidental finding in a 2‐year‐old boy with global mental retardation to acute neurological deficits caused by recurrent bleedings (age of onset: 1–32 years, Table 1). The mean age of the parents at birth of the index probands was 31.8 years (maternal range: 24–38 years, paternal: 20–39).

### Amplicon deep sequencing (ADS) identified a postzygotic *CCM3* mutation

The DNA samples isolated from blood lymphocytes of each de novo case were reanalyzed by ADS to distinguish between constitutional and postzygotic mutations after cut‐off values for allelic ratios of constitutional variants had been established (lower cut‐off ADS: 0.39; Fig. S1). The mean target sequencing depth was 900× (min: 93×; max: 1865×). Allelic read ratios (range: 41–55%) indicated constitutional status in five of the six de novo cases (P2–P6, Table 1). The respective mutation could not be found in any of the parental blood samples by ADS (Table S1).

**Figure 1:**
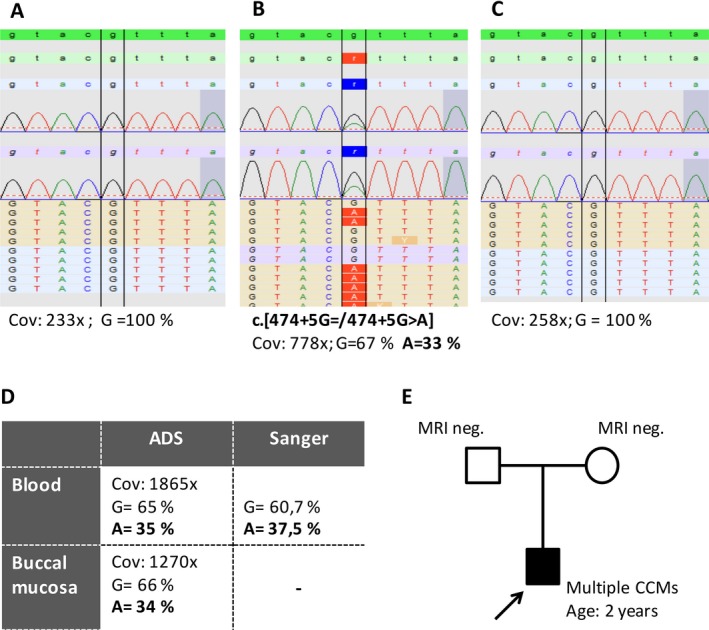
Mosaic *CCM3* splice site mutation (NM_007217.3; ENST00000392750.6) identified in P1. Lymphocyte DNA of the father (A), index (B), and mother (C) were used for HaloPlex target enrichment. Pseudo‐electropherograms representing alignments in SeqNext software are depicted. ADS and Sanger sequencing of lymphocyte DNA and/or DNA from buccal mucosa of the index case showed comparable results (D). The pedigree of the family is shown in (E). Cov = coverage; MRI = magnetic resonance imaging; neg. = negative.

However, the variant allelic read ratios in ADS (coverage: 1865×) and HaloPlex enrichment (778×) for the *CCM3* splice mutation found in the lymphocyte DNA of case P1 (c.474+5G>A) showed a significant deviation from the expected value of a constitutional variant (ADS: 35%, *Z*‐score = −2.86; HaloPlex: 33%, *Z*‐score = −3.18; Fig. 1B,D and Table 1). ADS of DNA isolated from buccal mucosa also indicated mosaicism (Fig. 1D). These results were reproducible in independent library preparations and sequencing runs. Different PCR primers were used to exclude a possible amplification bias. The identified sequence variant had previously been described in the literature as pathogenic (Liquori et al. 2006; Shenkar et al. 2015) and the suspected splice defect was verified by Liquori et al. using transcript analysis (Liquori et al. 2006). The mutation was excluded in both parental blood samples by deep sequencing using HaloPlex enrichment (Fig. 1A,C).

### Postzygotic deletion of the entire *CCM3* gene

**Figure 2:**
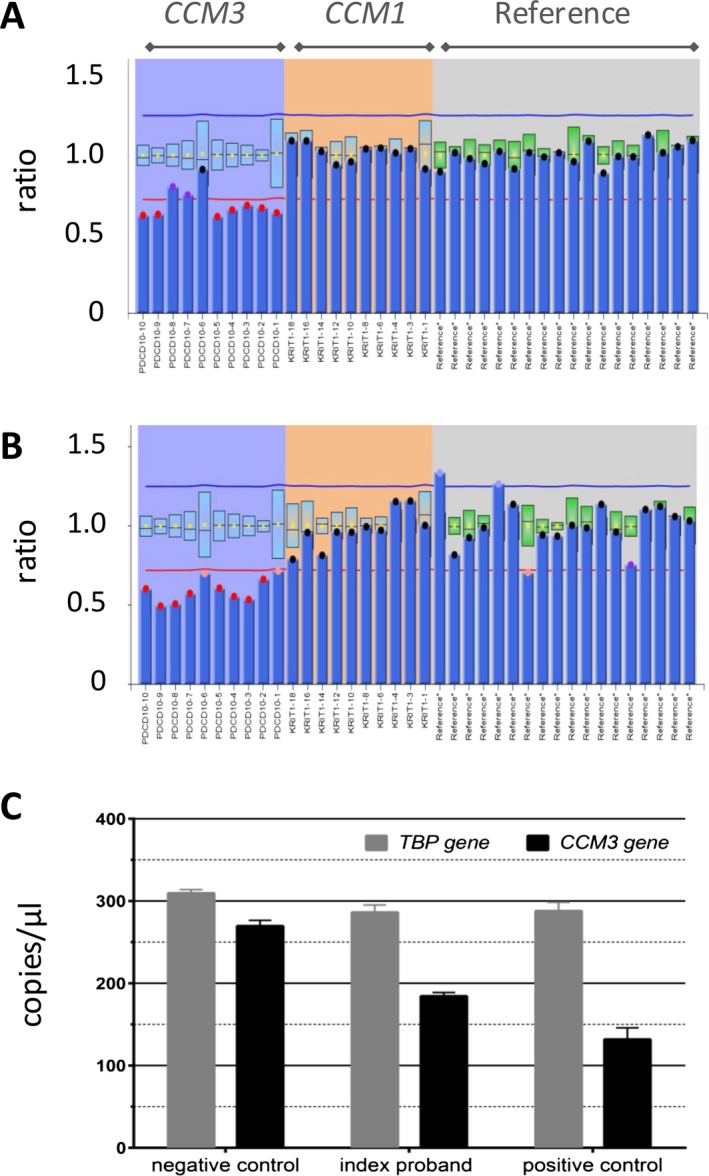
*CCM3* deletion (NM_007217.3; ENST00000392750.6) identified by MLPA analysis in lymphocyte DNA (A) and buccal mucosa DNA (B) of case P7. The height of the columns represents the dosage of the segments. The allele dosages of the deleted probes were hardly below the cut‐off value (<0.75) in the blood. Absolute quantification using digital PCR also implicated mosaicism (C). A carrier of a familial *CCM3* deletion and a mutation‐negative proband served as controls.

If parents are not available for genetic testing, results of molecular analyses have to be interpreted with even greater care. Using lymphocyte DNA, slightly decreased allele dosages (mean ratio after normalization: 0.69) below the lower cut‐off value (<0.75) were observed for all *CCM3*‐specific probes in MLPA analyses of case P7 (Fig. 2). These results were reproducible using independent blood as well as buccal mucosa samples and could be verified in different laboratories (data not shown). As parental blood samples were not available to confirm the suspected de novo event, a digital PCR assay was applied to determine the *CCM3* allele dosage with a second method (Fig. 2C). Equal DNA amounts were used for the *TBP* gene (TATA box‐binding protein) as reference and for positive and negative controls. Taken together, these results led to the assumption of mosaicism in case P7 with a fraction of about 30% of all *CCM3* alleles carrying the deletion.

## Discussion

Twenty *CCM3* (Bergametti et al. 2005; Liquori et al. 2006; Riant et al. 2013; Cigoli et al. 2014; Shenkar et al. 2015) and two *CCM1* de novo mutations (Lucas et al. 2001; Stahl et al. 2008) have previously been described in isolated CCM cases. Notably, one of these led to the identification of *CCM3* as disease‐associated gene (Bergametti et al. 2005). However, the prevalence of de novo mutations may still be underestimated since parents are not always available for genetic testing. The relatively high number of three *CCM3,* two *CCM1*, and one *CCM2* de novo point mutations as well as one additional postzygotic *CCM3* deletion in our series of isolated CCM cases (7/60; 11.6%) indicates that the prevalence of de novo events may be higher than previously thought. The predominance of *CCM3* de novo mutations that have previously been reported or newly identified in our cohort may also imply that *CCM3* is more prone to de novo mutational events. Interestingly, *CCM3* mutations are known to be associated with an earlier age of manifestation and a higher risk of hemorrhages (Riant et al. 2013; Spiegler et al. 2014; Shenkar et al. 2015). Aside from constitutional variants, somatic mutations in cavernous lesions of isolated CCM probands have been reported (McDonald et al. 2014). These are hardly ever detectable by conventional sequencing of lymphocyte DNA.

The interpretation of molecular diagnostics for isolated CCM cases can be challenging. In the context of genetic counseling, the identification of low‐level mutations as well as the discrimination between high‐level mosaicism and constitutional mutations is important to give a reliable risk assessment for CCM patients and their families. In accordance with previous reports (Jamuar et al. 2014), our data demonstrate that for this discrimination, the specificity of conventional sequencing is insufficient in most cases. Ultradeep sequencing might be useful for this purpose. Nevertheless, the cut‐off values of the expected allelic ratios for true heterozygous variants have to be carefully established with a known set of germline mutations for the respective technique (Acuna‐Hidalgo et al. 2015) (Fig. S1). Using NGS of DNA from blood lymphocytes and buccal mucosa, we have identified a likely mosaic mutation (P1). As comparable ratios of the alternate allele were found in the derivatives from different germ layers (mesoderm and ectoderm), an early postzygotic event rather than a selection against mutant cells in the blood (Huisman et al. 2013) was assumed. Interestingly, the *CCM3* mutation had previously been reported as a de novo mutation in an independent CCM case (Liquori et al. 2006).

No significant difference in the phenotype (age of manifestation, bleeding history, number of CCMs) was observed upon comparison of de novo cases carrying constitutional mutations (P2–P6) and postzygotic mutations (P1, P7). Although the mutations were not identified in any of the parental blood samples of constitutional mutation carriers, gonadal mosaicism cannot be excluded and has to be considered.

In conclusion, we recommend a stepwise approach for obvious de novo mutations. In the first step, amplicon deep sequencing should be applied for the index case to search for postzygotic mutations, because the recurrence risk for the parents and the possibility of siblings to be a mutation carrier as well are negligible in these cases. As it is well known that the majority of constitutional de novo mutations originate in the parental germline, high‐sensitivity ultradeep sequencing of parental blood and maybe even of paternal sperm samples might be warranted for the future as a second step to distinguish between low level and gonadal mosaicism.

## Conflict of Interest

The authors declare no conflicts of interest.

## Supporting information


**Figure S1.** Distribution of alternate allele read ratios of inherited heterozygous variants identified by amplicon deep sequencing (A) or HaloPlex enrichment (B).
**Table S1.** Deep sequencing performed for parents of index cases with de novo mutations.
