# Supplementary material for: Constitutional de novo and postzygotic mutations in isolated cases of cerebral cavernous malformations
Source: Mol Genet Genomic Med. 2016 Dec 20;5(1):21–7. doi: 10.1002/mgg3.256 (PMC5241208; doi:10.1002/mgg3.256)
Supplement: Supplementary file 1 — Figure S1. Distribution of alternate allele read ratios of inherited heterozygous variants identified by amplicon deep sequencing (A) or HaloPlex enrichment (B). Table S1. Deep sequencing performed for parents of index cases with de novo mutations. [file MGG3-5-21-s001.pdf]

Supplementary Data

Material and Methods

Assessment of expected allelic read ratio for constitutional variants

Following the recommendations of Acuna-Hidalgo et al. (1), two independent data sets were sequenced with ADS or with Haloplex enrichment to assess the expected allelic read ratios of constitutional variants in the respective technique (ADS: Mother-daughter-duo; Haloplex enrichment: Father-daughter-duo). The study design was approved by the local ethics committee of the University Medicine Greifswald (registration number: BB 047/14) and all probands participated with written informed consent according to the German Gene Diagnostics Act. Genomic DNA was isolated from blood lymphocytes using NucleoSpin® Blood L Kit (Macherey-Nagel, Düren, Germany). A constitutional status was assumed for heterozygous variants of the child that were also present in heterozygous or homozygous state in the parental DNA sample. Only variants that have been previously reported in dbSNP (build 142) were included.

Reference:

1. Acuna-Hidalgo R, Bo T, Kwint MP et al. Post-zygotic Point Mutations Are an Underrecognized Source of De Novo Genomic Variation. Am J Hum Genet 2015: 97: 67-74.

Fig. S1. Distribution of alternate allele read ratios of inherited heterozygous variants identified by amplicon deep sequencing (A) or Haloplex enrichment (B). The mean allele ratio is indicated by a green (A) or red line (B); ± 2 standard deviations (SD) are indicated by dotted lines. n= number of variants.

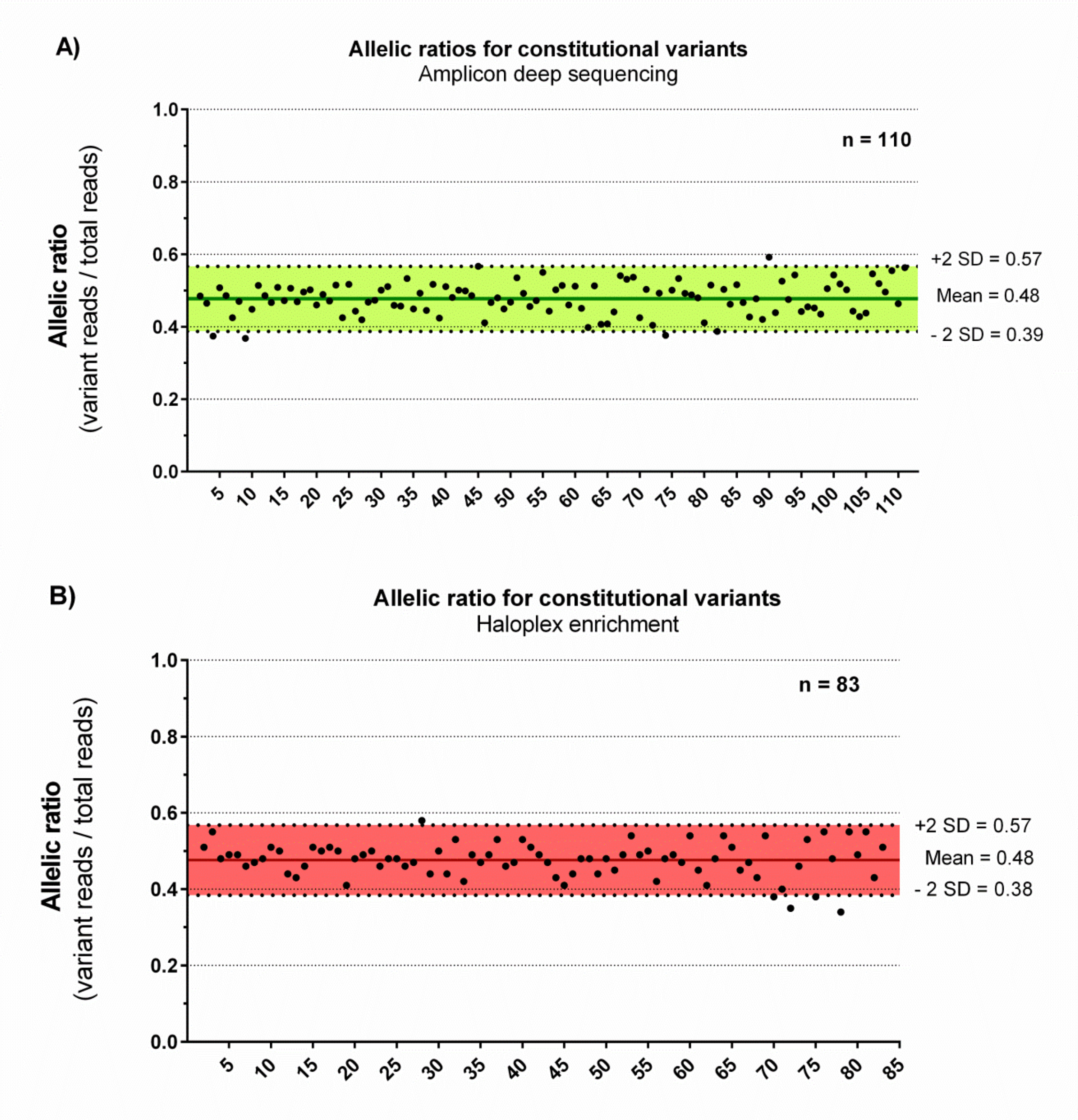

Table S1. Deep sequencing performed for parents of index cases with *de novo* mutations

Proband	Variant found in the index case	Father		Mother	
		Allelic ratio	Coverage	Allelic ratio	Coverage
P1	*CCM3:* c.474+5G>A	**0 %***	233x*	**0 %***	258x*
P2	*CCM2:* c.563_564dupGG	**0 %**‡	2316x‡	**0 %**‡	2041x‡
P3	*CCM3:* c.395+1G>A	**0 %**‡	437x‡	**0 %**‡	89x‡
P4	*CCM1:* c.1660_1678del	**0 %**‡	1842x‡	**0 %**‡	912x‡
P5	*CCM1:* c.1660_1678del	**0 %**‡	1533x‡	**0 %**‡	1207x‡
P6	*CCM3:* c.391delA	**0 %**‡	1058x‡	**0 %**‡	151x‡
P7	*CCM3:* deletion of the entire gene	n.d.		n.d.	

* assessed by Haloplex enrichment; ‡ assessed by amplicon deep sequencing; n.d. = not done
